# Differences in functional trait responses to elevation among feeding guilds of Aculeata community

**DOI:** 10.1002/ece3.9171

**Published:** 2022-08-04

**Authors:** Kazushige Uemori, Toshiharu Mita, Takuo Hishi

**Affiliations:** ^1^ Department of Agro‐environmental Sciences, Graduate School of Bioresource and Bioenvironmental Sciences Kyushu University Fukuoka Japan; ^2^ Department of Bioresource Sciences, Faculty of Agriculture Kyushu University Fukuoka Japan; ^3^ Department of Agro‐environmental Sciences, Faculty of Agriculture Kyushu University Fukuoka Japan; ^4^ Kyushu University Forest Fukuoka Japan

**Keywords:** Boreal forest, climate change, elevational gradient, feeding guild, functional trait, hymenoptera

## Abstract

The response of communities to environmental change is expected to vary among feeding guilds. To evaluate the response of guilds to environmental factors without considering the taxonomic specificities, it is useful to examine Aculeata bees and wasps, which consist of closely related taxa including different guilds, pollinators, predators, and parasitoids. In this study, we evaluated changes in species diversity (SD) and functional traits of each feeding guild along an elevational gradient in a boreal forest in northern Japan. We used yellow pan traps to collect Aculeata bees and wasps at 200–1600 m above sea level. We investigated six functional traits (trophic level, seasonal duration, body size, elevational range, nesting position, and soil dependency) and the horizontal distribution of the species. The SD of all Aculeata, predators, and parasitoids decreased with an increase in elevation; however, the SD of pollinators did not show any specific trend. Although the functional trait composition of all Aculeata species did not show any trend, that of each feeding guild responded to elevation in different ways. Pollinators increased in body size and showed a decrease in seasonal duration with increasing elevation, suggesting that tolerance and seasonal escape from physical stress at high elevations are important for shaping pollinator communities. Predators increased their elevational range and the proportion of above‐ground nesting species increased with increasing elevation, suggesting that the ability to live in a wider range of environments and avoid unsuitable soil environments at high elevations might be important. Parasitoids changed their hosts and displayed variable traits with increasing elevation, suggesting that brood parasitoids have difficulty in surviving at high elevation. The traits for each guild responded in different ways, even if they were dominated by the same environmental factors. Our findings imply that differences in the responses of functional traits would produce different community assembly patterns in different guilds during further climate change.

## Graphical abstract

The response of communities to climate change is expected to vary among feeding guilds. We found that functional trait composition of each feeding guild in Aculeata bees and wasps community responded to the elevational gradient in different ways. This differences can produce different community assembly patterns in different guilds during further climate change.

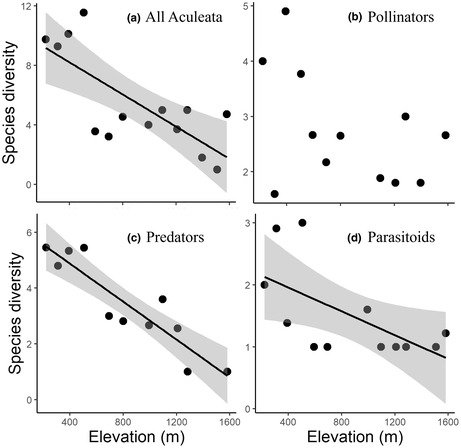

## 1 INTRODUCTION

Understanding the impact of environmental change on communities remains an unresolved challenge in community ecology. Elevational gradients have been used as a model of environmental change in many studies because they produce sympatric environmental gradients (Beck et al., 2010; Hodkinson, 2005). Recently, trait‐based approaches have been developed in both plant and animal community studies to quantify individual and species characteristics that are filtered by the environment to assess changes in community structure in response to environmental change (de Bello et al., 2021; McGill et al., 2006; Moretti et al., 2017). However, because animals have a wide range of traits, analysis using common traits is difficult unless the taxa groups are closely related.

Feeding guilds grouped by food resource and foraging type have different responses to the environment (Berg et al., 2010; Fontana et al., 2020; Pilliod & Rohde, 2016). In a multi‐trophic study, species richness (SR) patterns along an elevational gradient were different in each guild (Fontana et al., 2020). Different guilds showed marked differences in their sensitivity to temperature change and potential dispersal capacity (Berg et al., 2010). These differences in response to environmental change can lead to disruptions in community interactions, which can cause failures in the stability of biodiversity and the provision of ecosystem functions and ecosystem services (de Bello et al., 2021). However, most animals do not have multi‐guilds within closely related taxa groups, making it hard to separate guild and taxon characteristics. Therefore, differences in patterns between guilds and the mechanisms dominating each guild community are difficult to infer from analysis based on common traits.

Aculeata bees and wasps are represented by a large number of species and multi‐feeding guilds. They are highly correlated with the diversity of plants and arthropods (Duelli & Obrist, 2003; Fabian et al., 2014; Guo et al., 2021), and therefore are useful biological indicators (Brock et al., 2021). They also include three guilds with different foraging types—pollinators, predators, and parasitoids—in a closely related taxonomic group (three superfamilies: Apoidea, Chrysidoidea, and Vespoidea; Aguiar et al., 2013). Their similarity facilitates the use of common traits and allows the observation of guild‐specific responses without taxonomic features.

Different responses of functional traits to environmental gradients are expected among feeding guilds of Aculeata communities. Increased abiotic environmental stresses, such as lower temperatures and higher wind speeds with increasing elevation, result in trait responses such as increased body size and increased underground nesting of pollinator (Hoiss et al., 2012; Peters et al., 2016). The abiotic stress also increases elevational range, which promotes stress tolerance of species (Hoiss et al., 2012; McCain, 2009; Rapoport, 1982). Pollinators are also influenced by phenological factor, such as seasonality (Macgregor et al., 2019). Increased seasonality reduces seasonal duration time and leads to seasonal segregation (Macgregor et al., 2019; Randall et al., 1981; Uemori et al., 2021). Particularly in the alpine zone, the seasonal duration time of pollinators is linked to the limited flowering period (Kudo, 2014). Pollinators are predicted to respond to abiotic and phenological factors (Hoiss et al., 2012; Pilliod & Rohde, 2016).

Although there are fewer studies of the traits of predators and parasitoids compared to pollinators, their communities are expected to depend on the occurrence and attributes of diverse prey (Fornoff et al., 2021; Mayr et al., 2020). The trophic level shrinks in less productive environment in high mountainous area (Uemori et al., 2021). The extent to which predator and parasitoid communities utilize soil animals has rarely been quantitatively discussed. However, species using soil animals may be more tolerant to environmental change because the biomass of soil animals does not change with environmental change, such as forest conversion (Hasegawa et al., 2009; Salamon et al., 2008; Scheu et al., 2003). Especially at high elevation in boreal forests, the relative ratio of soil animal‐using species in the predator and parasitoid community is expected to increase, as forest degradation and dwarfing reduce above‐ground resources. On the other hand, the body size of predator and parasitoid is more affected by prey and host than changes related to tolerance due to the abiotic environment. Although predators compensate for the small size of their prey with numbers, the body size of parasitoids using a single individual is more affected by the body size of their host. Also, although pollinator‐plant phenology is quickly mismatched (Stemkovski et al., 2020), predator–prey and parasitoid‐host phenology are somewhat longer and therefore more tolerant of climate change (Damien & Tougeron, 2019). Predators and parasitoids are expected to be less affected by seasonality. Therefore, the supply of resources is more important than the abiotic and phenological factors for predators and parasitoids.

This study aimed to clarify whether the pattern of species diversity (SD) and trait response differs between feeding guilds in an Aculeata community along an elevational gradient in a boreal forest in eastern Hokkaido, northern Japan. Each guild could potentially have a different combination of relationships between environmental factors and the functional traits that drive communities. We predicted that pollinators are affected by abiotic factors with an increase in body size, elevational range, and below‐ground nesting species and phenological factors with decrease in seasonal duration time; predators and parasitoids are affected by the supply of resources factor with decrease in trophic level and increase in dependence on soil animals.

## 2 MATERIALS AND METHODS

### 2.1 Study site

**FIGURE 1:**
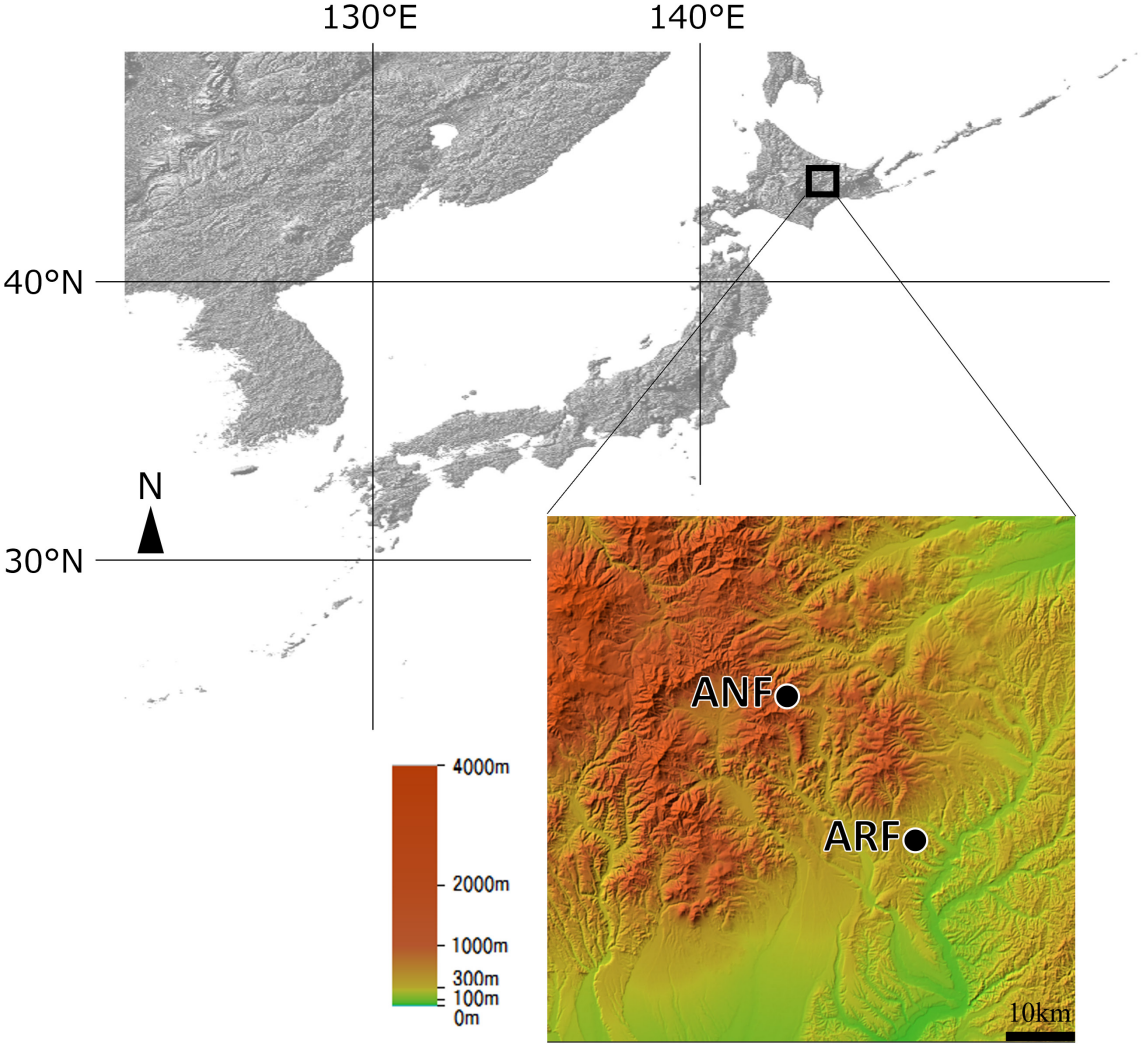
Locations of the study sites of the Ashoro Research Forest (ARF) and the Ashoro‐cho National Forest (ANF).

The study was carried out in old secondary and natural forests in the Ashoro Research Forest, Kyushu University (43°18′N, 143°31′E), and Mt. Kumaneshiri‐dake, the Ashoro‐cho National Forest (43°31′N, 143°15′E). These forests were located in the East Taisetsu Mountains (Figure 1, see Uemori et al., 2022 for full detail). We set 14 sites at different elevations from 223 m above sea level (a.s.l.) to 1581 m a.s.l. in these areas (Table 1, see Uemori et al., 2022 for full details). The lower elevation areas (223, 312, and 392 m a.s.l.) in the Ashoro Research Forest support deciduous broad‐leaved trees, such as *Quercus crispula* and *Tilia maximowicziana*, with *Sasa nipponica* as the understory vegetation. The middle elevation areas (507, 594, 695, 800, 993, 1097, and 1209 m a.s.l.) of this study on Mt. Kumaneshiri‐dake support a mixed forest dominated by *Alnus alnobetula*, *Betula platyphylla*, *Abies sachalinensis*, *Picea jezoensis*, and *Picea glehnii*, with *Sasa senanensis* as the understory vegetation. Higher elevation areas (1284 and 1396 m a.s.l.) are dominated by dwarfed *Betula ermanii* and *Acer ukurunduense*, with moss and fern communities as the understory vegetation. The top elevation areas (1509 and 1581 m a.s.l.) are dominated by *Pinus pumila,* with *Calamagrostis* sp. as the understory vegetation. The annual mean temperature was 6.22°C at 90 m a.s.l. in Ashoro from 2001 to 2017 (AMeDAS data from the Japan Meteorological Agency). The mean annual precipitation in Ashoro from 2001 to 2017 was 828 mm (AMeDAS data from the Japan Meteorological Agency, Tokyo). Snow cover extended from November to May, with the mean annual cumulative snowfall of 515 cm in 2016–2017 (AMeDAS data from the Japan Meteorological Agency).

**TABLE 1:** Site description includes elevation, dominant tree species, and understory vegetation

Elevation (m)	Dominant tree	Understory vegetation
223	*Fraxinus mandshurica, Ulmus davidiata*	*Sasa nipponica*
312	*Quercus crispula, Betula maximowicziana, B. platyphylla, Acer pictum, Maackia amurensis, Cerasus sargentii, C. maximowiczii*	*Sa. nipponica*
392	*Q. crispula, Tilia maximowicziana, Ac. pictum, B. maximowicziana, Alnus hirsuta*	*Sa. nipponica*
507	*Abies sachalinensis, Al. hirsuta, B. ermanii, Ac. ukurunduense*	*Sa. senanensis*
594	*Ab. sachalinensis, Picea glehnii, Al. hirsuta, B. ermanii*	*Sa. senanensis*
695	*Ab. sachalinensis, Al. hirsuta, B. ermanii*	*Sa. senanensis*
800	*Ab. sachalinensis, Al. hirsuta, B. ermanii*	*Sa. senanensis*
993	*Ab. sachalinensis, Picea glehnii, B. ermanii, Ac. ukurunduense, Sorbus commixta*	*Sa. senanensis*
1097	*Ab. sachalinensis, P. glehnii, B. ermanii, Ac. ukurunduense, So. commixta, Rhododendron multiflorum*	Fern spp.
1209	*Ab. sachalinensis, Ac. ukurunduense, So. commixta, R. multiflorum*	Moss & fern spp.
1284	*Ab. sachalinensis, P. jezoensis, B. ermanii, Ac. ukurunduense, So. commixta, Weigela middendorffiana*	Moss & fern spp.
1396	*Ab. sachalinensis, P. jezoensis, B. ermanii, Ac. ukurunduense, So. commixta, W. middendorffiana, C. nipponica*	Fern spp.
1509	*B. ermanii, Al. alnobetula, So. commixta, W. middendorffiana, R. multiflorum*	Fern & *Calamagrostis* spp.
1581	*Pinus pumila, B. ermanii, Al. alnobetula, So. commixta, P. glehnii, R. diversipilosum, Lonicera alpigena*	*Calamagrostis* spp.

### 2.2 Data collection

Yellow pan traps (15 cm in diameter) were used to collect Aculeata bees and wasps. Each pan was filled with approximately 150 ml of water, with a few drops of dishwashing detergent as a surfactant.

At each elevation site, we set up five plots under different tree individuals' canopies (at least 5 m apart). Four yellow pans were placed on the ground for each plot; therefore, 20 yellow pans were set up per elevation site. The samples in the yellow pans were collected in ethanol bottles at 48 h after installation. Because the prevalent species varied depending on the season, sampling was conducted three times in different seasons: 11–14 June (spring), 1–7 August (summer), and 16–27 August (autumn) in 2019. As almost Aculeata bees and wasps were not active in winter, no surveys were conducted from September to May (e.g. Kudo, 2014).

We sorted and identified the Aculeata from the collected samples at the species level. Formicidae were excluded as our sampling methodology was not suitable for this group. The identification of the bee and wasp species was conducted as per the guidelines of Tadauchi and Murao (2014) and Terayama and Suda (2016), respectively. Family groups followed Aguiar et al. (2013). All the species were divided into three guilds: pollinators, predators, and parasitoids, according to the guidelines (Tadauchi & Murao, 2014; Terayama & Suda, 2016). Voucher specimens were deposited at the Entomological Laboratory, Kyusyu University, Fukuoka, Japan (ELKU collection).

### 2.3 Functional traits and distribution index

We selected six functional traits expected to respond to changes in elevation: trophic level, seasonal duration, body size, elevational range, nesting position, and dependence on soil for food resources (Table 2; Table S1). (1) Trophic level: The trophic level was divided into three levels; pollinator bees (rank 1); primary predators feeding on Hemiptera, Lepidoptera, Orthoptera, and pollinator Hymenoptera (rank 2); hyper predators feeding Araneae, Diptera, and predator Hymenoptera (rank 3). The trophic level is expected to decrease with increasing elevation because lower temperatures reduce productivity (Uemori et al., 2021). (2) Seasonal duration: The seasonal duration was defined as the sum of the sampling seasons in which the species was collected. Because low temperatures and high seasonality at high elevations promote seasonal segregation, seasonal duration is expected to decrease with increasing elevation (Uemori et al., 2021). (3) Body size: Body size was the average of the literature data from Tadauchi and Murao (2014) (for bees) and Terayama and Suda (2016) (for wasps). For the four species that could not be identified, actual body lengths were measured. Body size is expected to increase with increasing elevation (Hodkinson, 2005; Hoiss et al., 2012 for bees; see Uemori et al., 2021 for all Aculeata). (4) Elevational range: The elevational range was defined as the difference between the lowest and highest elevations at which the species was collected. Species living at high elevations are expected to have a wider stress tolerance and, hence, a wider elevational range (Rapoport, 1982). Therefore, the elevational range of species is expected to increase with increasing elevation (Hoiss et al., 2012; McCain, 2009). (5) Nesting position: Two types of nesting positions during overwintering were identified: below‐ground nesting (including parasitoids of soil insects; rank 1) and above‐ground nesting (e.g. stems and wood; rank 2). The increase in belowground nesting with increasing elevation occurs because belowground nests provide the insects better protection against extreme climatic conditions (Hoiss et al., 2012). (6) Soil dependency: The dependence on soil for food resources was classified into two types: Aculeata feeding soil animals (detritivorous cycle; as in beetle larvae, flies, and non‐nesting spiders; rank 1) and Aculeata feeding flowers and above‐ground animals (herbivorous cycle; as in grasshoppers, caterpillars, and nesting spiders; rank 2) as a food resource for their young. To the best of our knowledge, there have been no previous studies on soil dependency in the Aculeata community. Extreme climatic conditions at high elevations and the reduction of aboveground structures as they approach the forest limit may increase soil dependency.

**TABLE 2:** Trait description includes value definition, expected change with increasing elevation, and reference for each trait

Trait	Definition of value	Expected change with increasing elevation	Reference
Trophic level	herbivorous primary predators hyper predators	Decrease	Uemori et al. (2021)
Seasonal duration	Total number of collecting seasons	Decrease	Randall et al. (1981); Macgregor et al. (2019); Uemori et al. (2021)
Body size	The mean of the literature data	Increase	Hodkinson (2005); Hoiss et al. (2012)
Elevational range	The highest elevation ‐ the lowest elevation	Increase	Rapoport (1982); Hoiss et al. (2012); McCain (2009)
Nesting position	below‐ground above‐ground	Decrease (increase below‐ground species)	Hoiss et al. (2012)
Soil dependence of food resource	herbivorous cycle detritivorous cycle	Increase (increase detritivorous cycle species)	
Distribution index	north of Hokkaido Hokkaido east Honshu west Honshu & Shikoku Kyushu Amami Is. & Okinawa Is. south of Yaeyama Is.	Decrease (increase northern species)	Uemori et al. (2021)

We also considered the species distribution bias, which reflects species dispersion and adaptation to temperature. The distribution index selected by Uemori et al. (2021) was used. The Japanese archipelago was divided into seven areas and ranked in order from the north area: (1) North of Hokkaido, (2) Hokkaido, (3) East Honshu, (4) West Honshu and Shikoku, (5) Kyushu (including Osumi islands), (6) Amami islands and Okinawa islands, and (7) Yaeyama islands and the Oriental region. The mean of the ranks of each species distribution was defined as the species distribution index. The index increased as the distribution of the species became more southerly (Table S1). In addition, distribution information for species was obtained from the data collected by Tadauchi and Murao (2014) and Terayama and Suda (2016). Finally, a distribution index of morphological species without a distribution record was applied to Region 2 (Hokkaido).

### 2.4 Statistical analyses

All samples were pooled for each elevation before analysis. The community was analyzed in two different ways: (1) all Aculeata and (2) three guilds (pollinator, predator, and parasitoid). For each elevational site, abundance, SR, and Simpson's index of SD were calculated. The functional diversity (FD) was also calculated for each trait based on the Rao index of diversity (Rao, 1982). Finally, the community‐weighted mean (CWM) was calculated for each trait. The CWM is weighted by the relative abundance of species with each trait value. The CWM is useful to summarize shifts in mean trait values within communities along environmental gradients (Ricotta & Moretti, 2011). In the calculations for pollinators, diversities and CWMs were not calculated because the trophic level and soil dependence were identical for all species. These values were calculated using the R package “vegan” (Oksanen et al., 2018) and ‘FD’ (Laliberte et al., 2014; Laliberte & Legendre, 2010). The relationships between elevation and community indices, such as abundance, SR, SD, FD, and CWMs, were analyzed using a linear regression model. All statistical analyses were performed using R version 3.5.0 for Windows (R Core Team, 2018). The diversity and CWM results for each elevation are shown in Table 3.

**TABLE 3:** Linear regression correlation coefficient between elevation and abundance, species richness (SR), species diversity (SD), functional diversities (FDs), and community‐weighted means (CWMs) of each functional traits in all Aculeata and each guild. A negative *r* value indicates a decrease in the response variable with increasing elevation. Trophic level, nesting position, and soil dependence in pollinator were not shown because the trait values were the same for all pollinator species. The significant values are shown bold.

	All Aculeata		Pollinator		Predator		Parasitoid	
	*r*	*p*	*r*	*p*	*r*	*p*	*r*	*p*
Abundance	−0.26	>.05	0.093	>.05	−0.34	>.05	−0.049	>.05
Species richness	**−0.73**	**.003**	−0.47	>.05	**−0.8**	**.003**	−0.53	>.05
Species diversity	**−0.75**	**.008**	−0.19	>.05	**−0.91**	**<.001**	**−0.61**	**.03**
FDs								
Trophic level	−0.44	>.05	‐	‐	**−0.75**	**.008**	**−0.69**	**.01**
Seasonal duration	−0.53	.05	**−0.73**	**.007**	**−0.64**	**.03**	**−0.70**	**.01**
Body size	−0.39	>.05	**0.71**	**.01**	−0.57	>.05	−0.47	>.05
Elevational range	−0.15	>.05	−0.16	>.05	0.031	>.05	−0.33	>.05
Nesting position	−0.29	>.05	‐	‐	**−0.72**	**.01**	−0.37	>.05
Soil dependence	−0.50	>.05	‐	‐	**−0.73**	**.01**	−0.37	>.05
Distribution index	−0.47	>.05	**0.70**	**.01**	**−0.67**	**.02**	−0.54	>.05
CWMs								
Trophic level	−0.24	>.05	‐	‐	0.35	>.05	**−0.74**	**.006**
Seasonal duration	−0.23	>.05	**−0.65**	**.02**	0.14	>.05	0.38	>.05
Body size	−0.13	>.05	**−0.62**	**.03**	−0.42	>.05	**−0.66**	**.02**
Elevational range	0.40	>.05	−0.24	>.05	**0.66**	**.03**	**0.65**	**.03**
Nesting position	−0.11	>.05	‐	‐	**0.73**	**.009**	−0.37	>.05
Soil dependence	0.50	>.05	‐	‐	−0.011	>.05	0.37	>.05
Distribution index	**−0.67**	**.009**	0.055	>.05	−0.43	>.05	**−0.64**	**.03**

## 3 RESULTS

In total, we collected 246 Aculeata individuals (68 in spring, 95 in summer, and 83 in autumn; 120 pollinators, 87 predators, and 39 parasitoids) and 64 species in 10 families (including 23 pollinator, 30 predator, and 10 parasitoid species) (Uemori et al., 2022). The most abundant species was *Lasioglossum problematicum* (38 individuals), which was collected at 392–1581 m a. s.l. and had the largest elevation range in this study.

**FIGURE 2:**
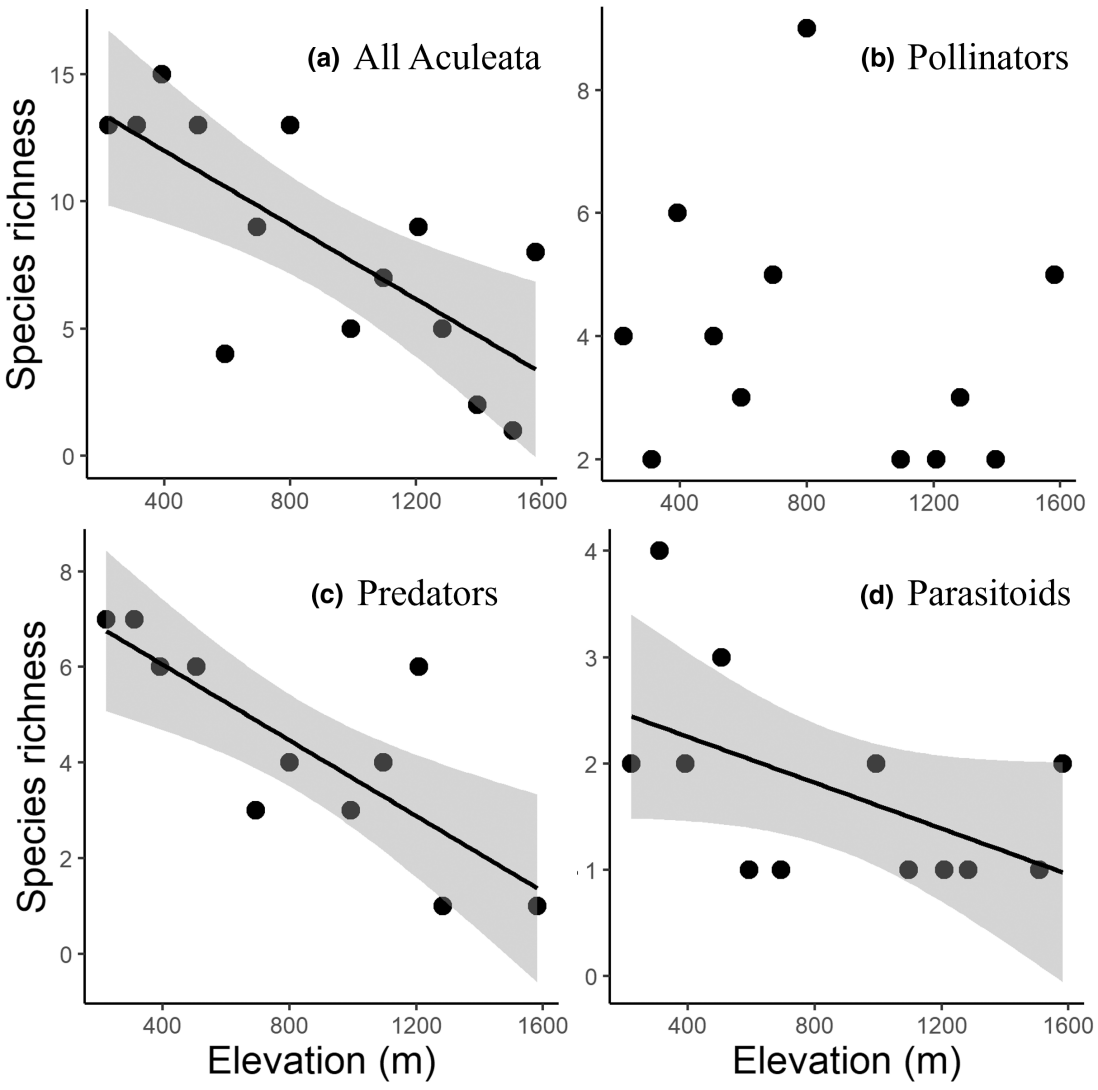
The relationship between elevation and species richness of all Aculeata and each feeding guild. The solid line and gray area indicate the result of linear regression and 95% CI, respectively. (a) All Aculeata (*r* = −0.73, *p* = .003); (b) pollinators (*p* > .05); (c) predators (*r* = −0.80, *p* = .003); and (d) parasitoids (*p* > .05).

**FIGURE 3:**
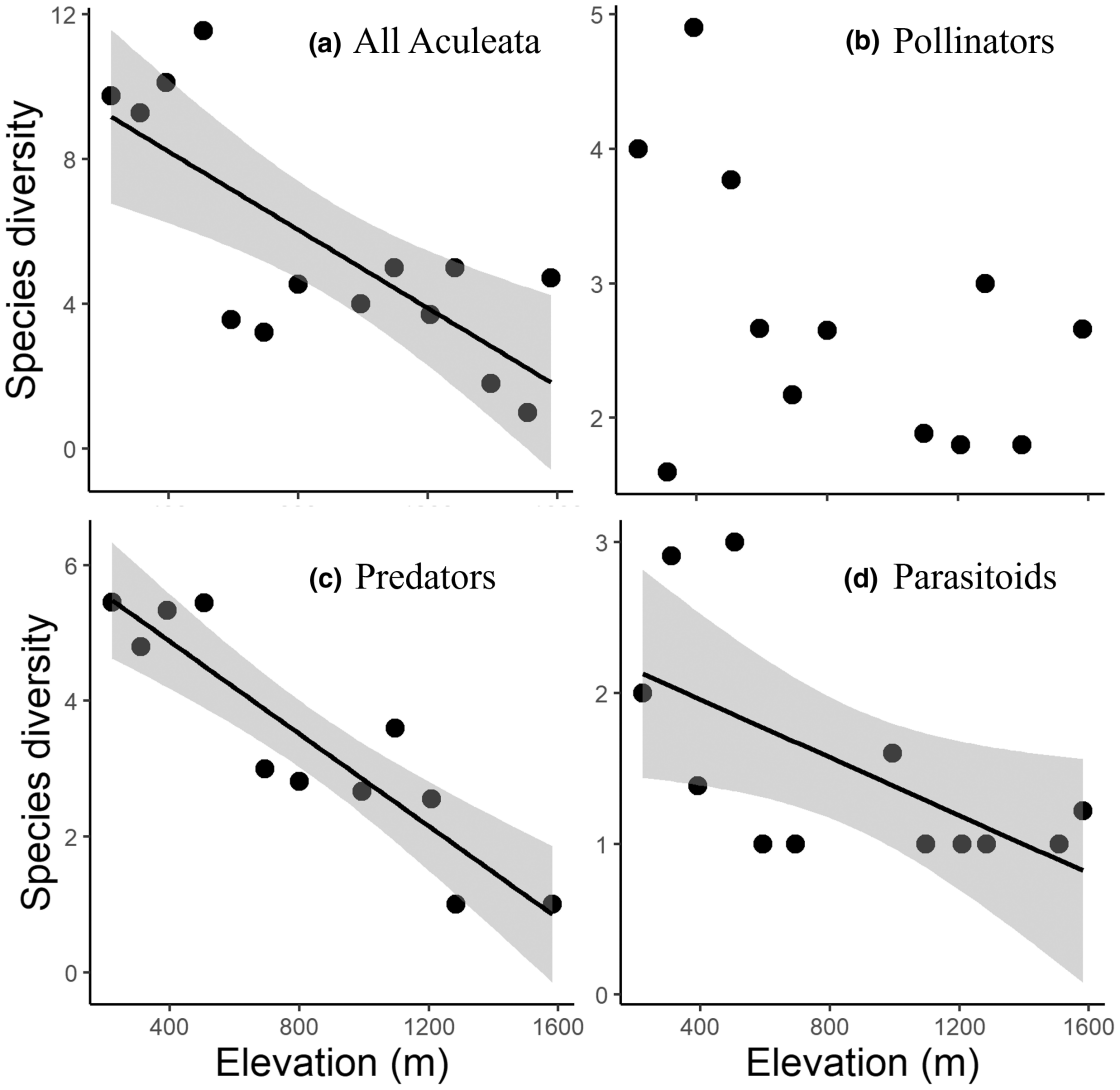
The relationship between elevation and species diversity of all Aculeata and each feeding guild, calculated using the Simpson's diversity index. The solid line and gray area indicate the linear regression result and 95% CI, respectively. (a) All Aculeata (*r* = −0.75, *p* = .008); (b) pollinators (*p* > .05); (c) predators (*r* = −0.91, *p* < .001); and (d) parasitoids (*r* = −0.61, *p* = .03).

**FIGURE 4:**
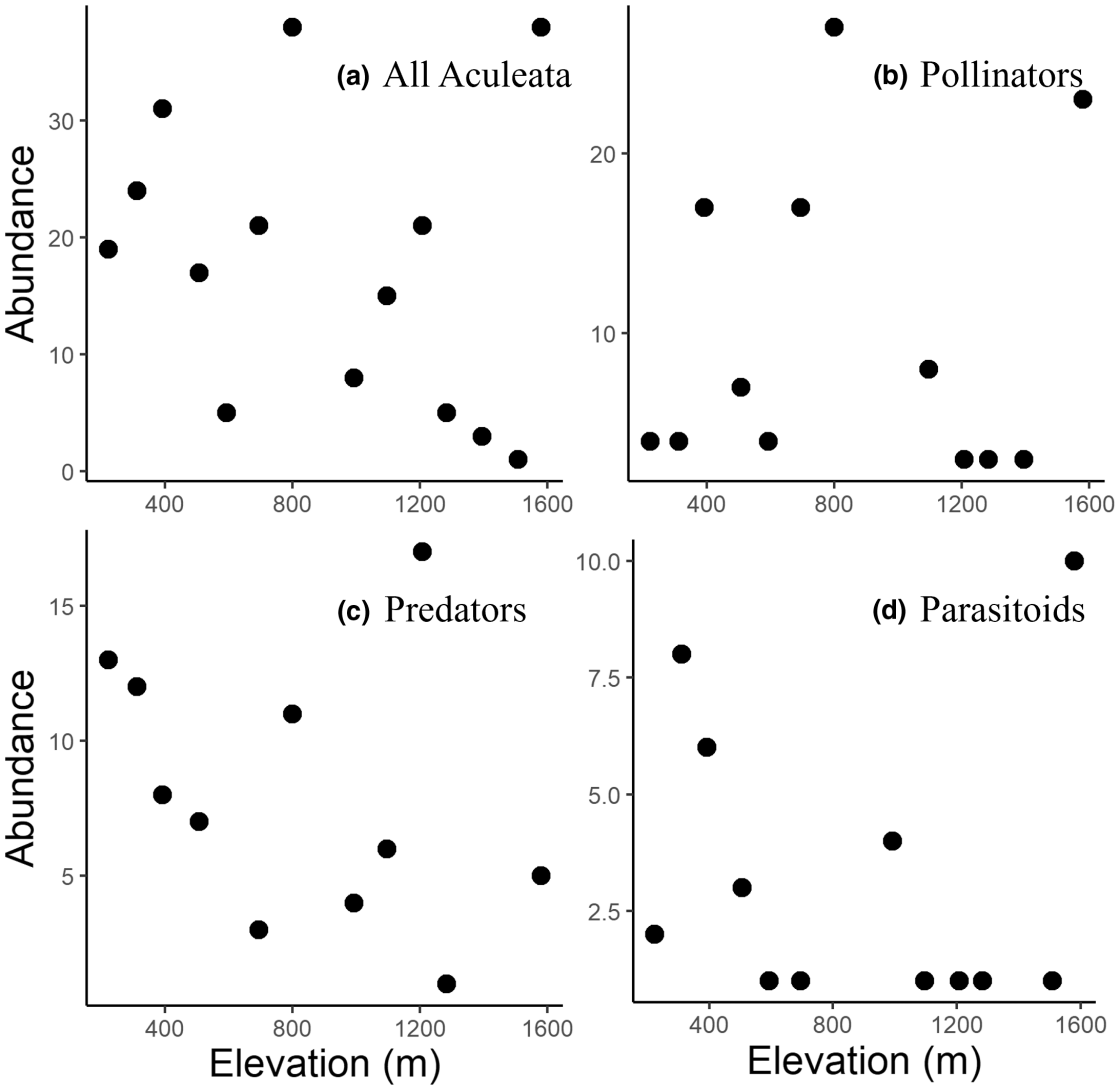
The relationship between elevation and abundance of all Aculeata and each feeding guild, calculated using the Simpson's diversity index. The solid line and gray area indicate the linear regression result and 95% CI, respectively. (a) All Aculeata; (b) pollinators; (c) predators; and (d) parasitoids (*p* > .05).

For all Aculeata communities, the SR decreased with increasing elevation (Table 3; Figure 2a, DF = 12). The SD based on Simpson's index also showed a significant linear decrease with increasing elevation (Table 3, Figure 3a). The FD of seasonal duration decreased with increasing elevation (Table 3). The CWM of the distribution index decreased with increasing elevation (Table 3). The abundance (Figure 4a), other FDs, and other CWMs did not show a significant pattern.

For the pollinator community, SR, SD, and abundance did not show significant patterns with elevation (Table 3; Figures 2b, 3b, 4b, DF = 10). The FD of seasonal duration decreased, and the FD of the body size increased with increasing elevation (Table 3). The diversity of the distribution index increased with increasing elevation (Table 3). The seasonal duration CWM decreased, and the CWM of body size increased with increasing elevation (Table 3). The other FDs and CWMs did not show a significant pattern along the elevation.

For the predator community, the abundance did not show a significant pattern with elevation (Table 3; Figure 4c, DF = 9). However, the SR decreased with increasing elevation (Table 3; Figure 2c), and the SD also decreased with increasing elevation (Table 3; Figure 3c). The FDs of trophic level, seasonal duration, nesting position, and soil dependence decreased with increasing elevation (Table 3). The CWM of elevational range increased with increasing elevation, whereas the CWM of the nesting position showed that above‐ground nesting species increased with increasing elevation (Table 3). The diversity of the distribution index decreased with increasing elevation (Table 3). At high elevations, the above‐ground nesting species (*Trypoxylon* spp.) were dominant, and few below‐ground nesting species were present. The other FDs and CWMs did not show a significant pattern along the elevation.

For the parasitoid community, abundance and SR did not show significant patterns with elevation (Table 3, Figures 2d, DF = 10). The SD decreased with increasing elevation (Table 3, Figure 3d). The FDs of trophic level and seasonal duration decreased with increasing elevation (Table 3). The CWMs of the trophic level and body size decreased, and the CWM of the elevational range increased with increasing elevation (Table 3). The CWM of the distribution index decreased with increasing elevation (Table 3). The lower elevations (below 507 m a.s.l.) were inhabited by brood parasitoid species (e.g. *Nysson trimaculatus* and *Sphecodes* spp., which attack bees and wasps), whereas the middle and higher elevations (above 507 m a.s.l.) were inhabited by koinobiont parasitoid species (*Anteon* spp., which attack leafhoppers).

## 4 DISCUSSION

We found that each guild showed different traits along an elevational gradient. Pollinators showed changes in body size and seasonal duration, suggesting that they were most affected by abiotic and phenological factors. Predators changed their elevational range and nesting position, suggesting that they were most affected by abiotic factors. The parasitoids changed their trophic level, body size, elevational range, and nesting position, suggesting that they were most influenced by abiotic and resource supply factors. We also found that different traits responded to each guild even if they were influenced by abiotic factor commonly.

Our finding that SD decreased with increasing elevation for all Aculeata is consistent with that of previous studies (Rahbek, 2005). Our site has a cold, dry climate like many previous study sites in Europe, where SD has been shown to decrease with increasing elevation (e.g. Corcos et al., 2018; Hoiss et al., 2012; Sydenham et al., 2015). Compared to Uemori et al. (2021), which was surveyed using the same sampling methods and elevational range in temperate forests, the SD in our study was lower. The climate at our site had the effect of reducing the diversity of Aculeata with increasing elevation. Alternatively, the low tree diversity in the area may have reduced the SD of Aculeata (e.g. Fabian et al., 2014; Guo et al., 2021). However, functional traits cannot sufficiently explain the mechanism controlling the assembly of all Aculeata communities, which are affected by environmental filtering and geographical conditions. The result of CWM for the distribution index suggests that the Aculeata community varies in its adaptation to climate along the elevational gradient. However, how they adapt is not clear from the characteristics examined in this study.

Contrary to the results of previous studies (Araújo et al., 2006; Hoiss et al., 2012; Sydenham et al., 2015), the SD of the pollinator community did not significantly change with elevation. However, the effects of temperature stress on several functional traits have been observed. The shorter seasonal duration period of high elevation species is an adaptation to the shorter flowering period owing to the strong seasonality of the alpine zone. The alpine spring community is unique, and pollinators are sensitive to flowering (Kudo, 2014; Kudo, 2016). Some species appear only during the spring season at high elevations (*Andrena lapponica* and *Andrena subopaca*). This suggests that the SD did not drop even at high elevations because of the presence of species specializing in the blooming season. The increase in body size with increasing elevation is an adaptation to temperature stress (Hoiss et al., 2012; Horne et al., 2017; Peters et al., 2016). Larger species can regulate their body temperature more effectively and fly at lower temperatures, thus improving their foraging ability (Peters et al., 2016). Their large bodies are an adaptation enabling them to take advantage of alpine plants that bloom during cooler periods. In addition, flower abundance affects the SD of pollinators (Araújo et al., 2006; Sydenham et al., 2015; Uemori et al., 2021; but see Hoiss et al., 2012). Although flower coverage was not measured in this study, flowering trees and grasses such as *Acer* spp. were present at all sites. Therefore, the number of flowers was not considered a limitation for SD.

Our findings for predator SD are in agreement with many studies on elevation (e.g. Corcos et al., 2018), and the same is true for our finding that predator richness increases with sites with warmer minimum winter temperatures (Pilliod & Rohde, 2016). The increase in the elevation range with increasing elevation indicates that highland predator species have broader environmental tolerance. According to Janzen (1967), these species have wider environmental tolerances and larger elevational ranges, owing to greater seasonality (McCain, 2009). Therefore, it is likely that species with high environmental tolerance would live in highland areas. The reason for the presence of few below‐ground nesting species at high elevations is unclear. One possible explanation is that high elevations have humid ground covered with mosses and ferns. In this situation, nesting in the soil may expose the insects to fungus and infection. Humidity increases the probability of parasite and pathogen spread (Fornoff et al., 2021; Hranitz et al., 2009). In addition, colder winter temperatures may indicate greater snow insulation for individuals buried just below the soil surface in pollinator communities (Pilliod & Rohde, 2016). The exact amount of snowfall at our sites was not measured, but the Tokachi region, where our site is located, is known to have low snowfall and deep soil freezing (Hirota et al., 2006). In areas with little snow cover, nesting above the ground, using dead wood instead of soil, maybe more favorable for survival.

The SR of the parasitoids decreased with increasing elevation as seen in our results and those of Morris et al. (2015), while some other studies have shown no change in SR along the elevation gradient (Hoiss et al., 2012; Maunsell et al., 2015). Some traits in parasitoids were strongly affected by differences in their hosts. Morris et al. (2015) showed that parasitoids and their host species at higher elevations were nested with species at lower elevations. This means that widely stress tolerant species living in low elevation were also present at high elevation. In contrast, the species composition had a high turnover along elevation in our study. There appear to be few northern species of parasitoid among Aculeata (e.g. Bethylidae and Tiphiidae, Terayama & Suda, 2016), which may explain the low number of individuals and richness collected in our study. The decrease in the trophic level and body size and the increase in above‐ground nesting species are explained by changes in the hosts with increasing elevation. The absence of brood parasitoids at higher elevations may be due to a decline in host bees and wasps. The SR of brood parasitoids using bees decreases with increasing elevation due to a decrease in the SR of their host bees (Mayr et al., 2020). By contrast, the koinobiont parasitoid species may have emerged at higher elevations because there was a suitable habitat for their host leafhopper. *Anteon* spp. usually parasitise leafhoppers (Cicadellidae), which feed on grass and tree leaves (Guglielmino et al., 2013). At high elevations, trees are dwarfed and have lower canopies. Thus, arboreal species may be trapped more easily. Nevertheless, *Anteon scapulare*, was abundant not only in dwarfed forests at high elevations but also at low elevations of 500 to 1200 m a. s.l., where the canopy was about 20 m high. This elevational range indicates that *Anteon* spp. at higher elevations are highly stress‐tolerant. The distribution index results suggest that the species can adapt to the climate at each elevation. Many questions remain regarding the diversity patterns of parasitoid insects (Santos & Quicke, 2011). It is suggested that community assembly mechanisms in parasitoids are complex, depending on the climate, environment, and host–parasitoid interaction in each case.

This study revealed that traits differ in response to factors dominated by community structure for each guild, even in closely related taxonomic groups. This finding suggests that as many traits as possible should be considered when comparing and clarifying the dominant factors for multiple guilds, even if they are expected to respond to the same dominant factor. The amount of resources, such as pollen, leafhopper, and spider, may give a clearer understanding of the mechanisms dominating each guild. In addition, the boreal forest, including our study site, is strongly affected by climate change (Bartomeus et al., 2011; IPCC, 2007; Walsh, 2014). The fact that guilds showing the same SD patterns respond differently to functional traits implies that differences in the responses of functional traits may in the future lead to different community assembly patterns for each guild as climate change progresses. Finding the dynamics of each guild will help to estimate the ecosystem functions that will be lost due to environmental change.

## AUTHOR CONTRIBUTIONS

**Kazushige Uemori:** Conceptualization (equal); data curation (lead); formal analysis (equal); funding acquisition (supporting); investigation (lead); methodology (lead); visualization (lead); writing – original draft (lead). **Toshiharu Mita:** Funding acquisition (supporting); investigation (supporting); supervision (supporting); writing – review and editing (equal). **Takuo Hishi:** Conceptualization (supporting); funding acquisition (lead); investigation (supporting); supervision (lead); writing – review and editing (equal).

## CONFLICT OF INTEREST

Authors declare no conflict of interests.

## Supporting information


Table S1

Figure S1

## Data Availability

The data that supports the findings of this study are available in the supplementary material of this article.
