# Supplementary material for: Differences in functional trait responses to elevation among feeding guilds of Aculeata community
Source: Ecol Evol. 2022 Aug 4;12(8):e9171. doi: 10.1002/ece3.9171 (PMC9353017; doi:10.1002/ece3.9171)
Supplement: Supplementary file 2 — Figure S1 [file ECE3-12-e9171-s001.docx]

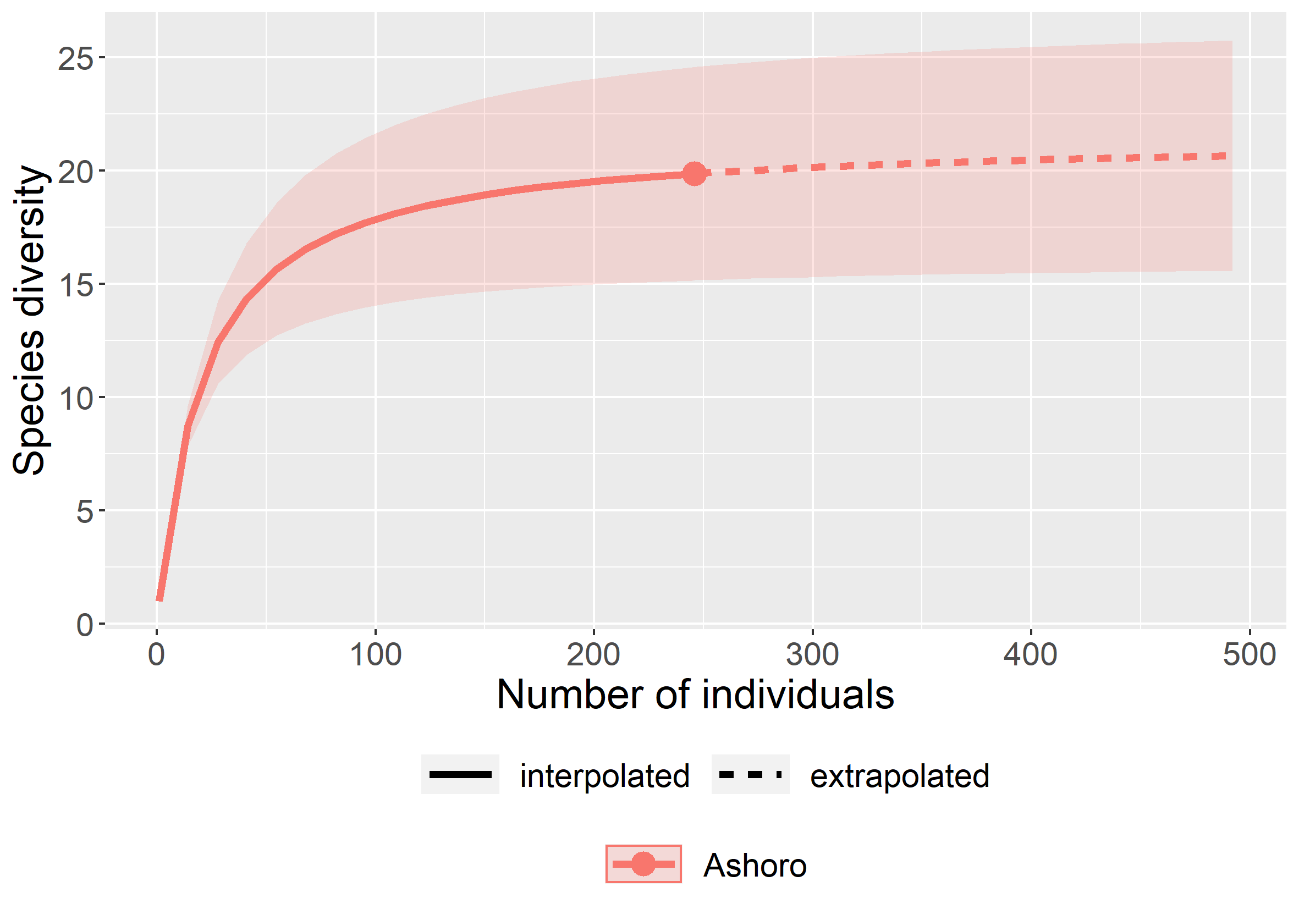


Figure S1: Rarefaction curve of species diversity based on Simpson’s index in all study site using iNEXT package (Chao et al., 2014; Hsieh et al., 2016). Solid line shows interpolated, and dotted line shows extrapolated.

**References**

Chao, A., Gotelli, N. J., Hsieh, T. C., Sander, E. L., Ma, K. H., Colwell, R. K., & Ellison, A. M. (2014). Rarefaction and extrapolation with Hill numbers: a framework for sampling and estimation in species diversity studies. Ecological Monographs, 84, 45–67. <https://doi.org/10.1890/13-0133.1>

Hsieh, T. C., Ma, K. H., & Chao, A. (2016). iNEXT: an R package for rarefaction and extrapolation of species diversity (H ill numbers). Methods in Ecology and Evolution, 7(12), 1451-1456. <https://doi.org/10.1111/2041-210X.12613>
